# Differential response of bovine mammary epithelial cells to *Staphylococcus aureus* or *Escherichia coli* agonists of the innate immune system

**DOI:** 10.1186/1297-9716-44-40

**Published:** 2013-06-11

**Authors:** Florence B Gilbert, Patricia Cunha, Kirsty Jensen, Elizabeth J Glass, Gilles Foucras, Christèle Robert-Granié, Rachel Rupp, Pascal Rainard

**Affiliations:** 1 INRA, UMR1282, ISP, F 37380, Nouzilly, France; 2 Université François Rabelais de Tours, UMR1282, ISP, F 37000, Tours, France; 3 The Roslin Institute, University of Edinburgh, Easter Bush Campus, Midlothian, Edinburgh EH25 9RG, United Kingdom; 4 INRA, UMR1225, IHAP, F 31076, Toulouse, France; 5 Université de Toulouse, INP, ENVT, UMR1225, IHAP, F 31076, Toulouse, France; 6 INRA, UR631, SAGA, F 31326, Castanet-Tolosan, France

## Abstract

Mastitis caused by *Escherichia coli* and *Staphylococcus aureus* is a major pathology of dairy cows. To better understand the differential response of the mammary gland to these two pathogens, we stimulated bovine mammary epithelial cells (bMEC) with either *E. coli* crude lipopolysaccharide (LPS) or with *S. aureus* culture supernatant (SaS) to compare the transcriptomic profiles of the initial bMEC response. By using HEK 293 reporter cells for pattern recognition receptors, the LPS preparation was found to stimulate TLR2 and TLR4 but not TLR5, Nod1 or Nod2, whereas SaS stimulated TLR2. Biochemical analysis revealed that lipoteichoic acid, protein A and α-hemolysin were all present in SaS, and bMEC were found to be responsive to each of these molecules. Transcriptome profiling revealed a core innate immune response partly shared by LPS and SaS. However, LPS induced expression of a significant higher number of genes and the fold changes were of greater magnitude than those induced by SaS. Microarray data analysis suggests that the activation pathways and the early chemokine and cytokine production preceded the defense and stress responses. A major differential response was the activation of the type I IFN pathway by LPS but not by SaS. The higher upregulation of chemokines (*Cxcl10*, *Ccl2*, *Ccl5* and *Ccl20*) that target mononuclear leucocytes by LPS than by SaS is likely to be related to the differential activation of the type I IFN pathway, and could induce a different profile of the initial recruitment of leucocytes. The MEC responses to the two stimuli were different, as LPS was associated with NF-κB and Fas signaling pathways, whereas SaS was associated with AP-1 and IL-17A signaling pathways. It is noteworthy that at the protein level secretion of TNF-α and IL-1β was not induced by either stimulus. These results suggest that the response of MEC to diffusible stimuli from *E. coli* and *S. aureus* contributes to the onset of the response with differential leucocyte recruitment and distinct inflammatory and innate immune reactions of the mammary gland to infection.

## Introduction

Mastitis is ranked as the top disease for dairy cattle on the basis of incurred economic losses [1,2]. *Escherichia coli* and *Staphylococcus aureus* are two major pathogens causing mammary infections of dairy ruminants. Most cases of *E. coli* mastitis are clinical and of short duration, in general less than 10 days, because the inflammatory reaction is usually able to clear the infection [3]. In contrast, *S. aureus* mastitis may manifest very diverse degrees of severity, from fulminating gangrenous mastitis with nervous systemic signs to mild local infection with only local signs [4]. In the cow, most strains are associated with infection that often leads to chronic mastitis lasting several months [4]. Experimentally induced *E. coli* mastitis is characterized by high concentrations of chemokines and cytokines in milk, whereas these inflammatory mediators, and in particular the chemokine CXCL8 and the cytokine TNF-α, are undetectable or in low concentrations in case of *S. aureus* mastitis [5,6]. Once they have entered the lumen of the mammary gland through the teat canal, these bacteria are able to replicate rapidly in milk. A rapid and robust response of the mammary gland is needed for the control of these fast-growing microbial invaders. Consequently, the prompt detection of pathogens by the innate immune system is of prime importance, and because mammary epithelial cells (MEC) are in the front line, they could play an important role as sentinels. Several studies have showed that MEC are poised to respond quickly to bacterial intrusion through the activation of several Pattern Recognition Receptors (PRR) by the so-called Microbe-Associated Molecular Patterns (MAMP). MEC are equipped with several sensors of bacteria, and they are able to react by producing mediators of inflammation and local defense [7-9]. Thus, an important research issue is to define how MEC play their role of sentinel of the mammary gland.

The main objective of our study was to characterize the differences in the response of MEC to *E. coli versus S. aureus* at the onset of infection, which could impact the triggering of the inflammatory and immune responses of the mammary gland.

A number of studies have shown that bovine MEC in culture are able to sense bacteria or bacterial products, and to react by up-regulating several sets of genes involved in the innate immune response [10-13]. Although a great deal of information has been gathered, the innate immune sensing of *S. aureus* or *E. coli* in the mammary gland and the induced immune responses are not completely understood. These processes are of crucial importance because they can lead to either effective antibacterial responses or harmful side-effects of inflammation. We set out to study the response of bovine MEC to components of *E. coli* and *S. aureus* with a view to uncovering some of the reasons that could account for the contrasted responses of the udder to these two pathogens.

Most of the studies that have shown that bMEC respond differently to *E. coli* and *S. aureus* were based on in vitro exposure of isolated bMEC to killed bacteria or purified bacterial MAMP [10,11,14,15]. It is known that the nature of the stimulus, i.e. live or killed bacteria or purified MAMP, elicits somewhat different responses from target cells [16,17]. These may reflect the sequential host-pathogen dialogue that occurs as bacteria begin to multiply in milk following intrusion into the lumen of the mammary gland. Bacteria are probably first detected by host sensors through the release of soluble and particulate factors which increase during bacterial stress, as it might be expected in the early stages of bacterial colonization of the mammary gland. These complex interactions which occur prior to the detectable onset of the inflammatory response are likely to determine the ultimate outcome in terms of pathogenic sequelae leading to acute or chronic mastitis. However, relatively little is known about the initial host response to these factors.

We chose to tackle the issue by using complex mixtures of bacterial components rather than live bacteria. In the mammary gland at the onset of infection, sentinel cells are confronted by bacterial bodies (mostly live bacteria), and by products secreted by bacteria. Turnover of peptidoglycan in the course of replication, active secretion of exoproteins (toxins, proteases) or shedding of surface proteins released by proteases (adhesins, other surface proteins) or other mechanisms such as extrusion of membrane particles are involved in the release of bacterial products [18,19]. Thus, the response of sentinel cells probably results from the combination of bacterial bodies and released diffusible products. However, when bacterial density remains low in the lumen of the mammary gland, the bacterial components shed precociously during bacterial proliferation, are likely to interact with more MEC than the bacterial bodies themselves. Therefore in this study we focused our interest on the early response of MEC to these bacterial factors. The objective was to mimic the onset of intramammary infection (IMI) when the epithelium lining initially makes contact with components of bacterial origin. During growth, *E. coli* bacteria do not shed great amounts of proteins or other soluble compounds, but they release many outer membrane vesicles (omv) [19]. These omv are complexes of LPS with proteins including lipoproteins. We used *E. coli* crude LPS as a commercial substitute for omv. In contrast to *E. coli*, *S. aureus* bacteria secrete a lot of proteins along with insoluble particles during growth. Culture supernatant from a mastitis-causing *S. aureus* (SaS) was taken in this study as a source of staphylococcal released bacterial products. To further our understanding of the pathways involved in these early events, we compared the responses of bMEC to crude LPS and to SaS by microarray analysis. We found that more genes were differentially expressed, and that the magnitude of expression was higher, after stimulation with LPS than with SaS. A major difference in the nature of the induced response was the activation of the type I IFN pathway by LPS, but not by SaS, with a concomitant overexpression of several genes involved in either the recruitment of mononuclear leucocytes or local defenses. The most affected activation and functional pathways also differed. Overall, these results strongly suggest that *E. coli* induces a more intense response associated with strong NF-κB stimulation and the recruitment of a wider repertoire of immune cells, whereas *S. aureus* interferes with cell DNA integrity and may induce a more restricted immune response involving the IL-17A pathway.

## Materials and methods

### Bacterial agonists of bMEC

Four *S. aureus* strains were initially selected to prepare SaS. Three of them (169.32, 628.24 and 644.15) were from our mastitis strain collection, and were originally isolated from subclinical cases of mastitis. Some characteristics of strains 628.24 and 644.15 are described elsewhere [20]. The fourth strain, Newbould 305 (N305), was originally isolated from a clinical case of bovine mastitis and subsequently used as a model organism to experimentally induce mastitis and study the inflammatory response of the mammary gland or the response of bMEC [5,21,22]. On sheep blood agar plates, this strain is strongly alpha and beta hemolytic. A few features of the four shortlisted bacterial strains are given in Table 1. To prepare culture supernatants, bacteria stored lyophilized or at -80°C were cultivated overnight in Brain Heart Infusion broth, then were grown overnight at 37°C in RPMI 1640/DMEM (1:1) (Gibco, Invitrogen, Carlsbad, CA, USA). Finally, 50 mL of RPMI 1640/DMEM was seeded with 0.5 mL of the overnight culture, and incubated at rest in a 165 mL flask for 8 h at 37°C, or for different durations as indicated in the Results section. Protein content of 8-h SaS was 40 μg/mL for strains N305 and 169.32, and 20 μg/mL for strains 628.24 and 644.15, as measured with a micro-BCA assay (Pierce, Thermo Scientific, Rockford, IL, USA).

**Table 1:** **Features of *S. aureus *bacterial strains shortlisted for stimulation of bMEC**

**Strains**	**Hemolysis phenotype**	**Agr type**	**Capsular type**	**Leucotoxin titer**	**LukS (ng/mL)**	**Hla (μg/mL)**
Newbould 305	hla+++ hlb+++	agr1	CP5	20	5	10
169.32	hla+++ hlb+++	agr2	CP8	120	50	5
628.24	hla- hlb-	agr1	CP5	10	0	0.6
644.15	hla- hlb-	agr1	CP5	5	0	0.6

*Hla* Alpha hemolysin, *hlb* Beta hemolysin, *CP* Capsular polysaccharide, *LukS* Small component of bi-component leucotoxins. Toxin titers and concentrations were measured in 8-h culture supernatants.

Crude LPS (phenol extract, *Escherichia coli* O55:B5, catalog reference L-2880) was from Sigma-Aldrich (St-Louis, MO, USA). Purified LPS from *E. coli* O111:B4 (ULPS), purified staphylococcal lipoteichoic acid (LTA), synthetic muramyl dipeptide (MDP), purified flagellin from *Salmonella enterica* serovar Typhimurium and C12-iE-DAP were from InvivoGen (Toulouse, France). Staphylococcal protein A (SpA) was from Gentaur (GENTAUR Europe BVBA, Brussels, Belgium) and recombinant Protein A from MBL (JM-6500B) (Clinisciences, Nanterre, France). The staphylococcal α-hemolysin was purified as previously described [23,24].

### Detection of MAMP with HEK293 cell lines

HEK-Blue™ reporter cells, which are stably transfected with multiple human genes from the TLR2 (HEK-Blue-2) or the TLR4 (HEK-Blue-4) pathways and with a reporter gene monitoring NF-κB activation, were purchased from InvivoGen. These cells were dispensed in 96-well plates (2 × 10^4^ per well) and cultured for 48 h. Then they were incubated with either LPS or SaS and HEK-Blue detection medium for 16 h. The presence of TLR2 or TLR4 agonists induced the reduction of the medium which turned blue. The blue color was quantified by measuring absorption at 650 nm. HEK293 cells stably transfected with either the human *TLR5* gene, *CARD4* gene (encoding the PRR NOD1) or *CARD9* gene (encoding NOD2) were from InvivoGen. They were used as HEK-Blue reporter cells, except that at the end of incubation with LPS or SaS (without HEK-Blue detection medium), the cell culture supernatant was collected and the CXCL8 content measured by ELISA as a readout. Purified agonists of PRR (InvivoGen) were used in parallel with LPS and SaS as positive and negative controls.

### Characterization of SaS

Staphylococcal cultures were centrifuged and supernatants were aseptically filtered on 0.2 μm filters and stored at -80°C. Aliquots were saved for SaS characterization. For each strain supernatant, protein concentration was determined with the Micro-BCA protein assay. The protein content was analyzed by SDS-PAGE on 12.5% polyacrylamide gel and ammoniacal silver staining. The presence of specific components in staphylococcal supernatants, such as LTA, SpA and α-hemolysin were revealed by immunoblotting using relevant antibodies, i.e. mouse monoclonal antibody ab12248 to LTA from Abcam (Cambridge, UK), chicken polyclonal antibody ab18598 to SpA from Abcam and an in-house rabbit polyclonal antibody to α-toxin.

Pilot experiments were carried out for *S. aureus* strain selection and SaS production. As staphylococcal strains can vary greatly in their ability to stimulate cellular responses [25], we compared the response of MEC to 4 *S. aureus* mastitis isolates, selected on the basis of their use in previous studies [5,20]. In order to use early culture supernatants, we determined the minimum culture duration necessary to obtain a sizeable quantity and variety of proteins. It appeared that for the 4 isolates 7 to 8 h of culture were necessary to yield a reproducible pattern of proteins as visualized by SDS-PAGE analysis (Additional file 1 and results not shown). The 8-h culture corresponded to the early stationary phase for the 4 strains (results not shown). The 30–40 kDa zone was particularly rich in bands which correspond to the range of molecular masses of hemolysins and other toxins. The total quantity of proteins in the 8-h SaS was 40 μg/mL for strains N305 and 169.32, and 20 μg/mL for strains 628.24 and 644.15. To partially compensate for this difference in concentration, bMEC were stimulated with 25% 8-h N305 or 169.32 SaS and 33% 8-h 628.24 or 644.15 SaS. After 8 h of exposure to SaS, CXCL8 concentrations were determined in cell culture supernatant and transcripts of TNF-α were measured in MEC extracts. Although MEC responded to all the SaS, the response to N305 SaS was the greatest for the two indicators (Additional file 1 and results not shown). Consequently, the 8-h N305 SaS was selected for the study.

### Depletion of LTA from SaS

In order to investigate whether LTA contributed to the activation of MEC, SaS LTA content was reduced by affinity adsorption by using Protein G Sepharose beads coated with the anti-LTA mAb (Abcam) as described [26]. Briefly, to deplete LTA from SaS bead pellet (50 μL) was incubated for 3 h at room temperature under slight agitation with 100 μg of monoclonal ab12248. After centrifugation at 150 × *g* and five washing steps with HBSS, the activated beads were incubated with SaS (500 μL) for 6 h at 4°C under continuous shaking. Then the beads were removed by centrifugation. Control SaS was prepared by incubating SaS with uncoated beads.

### MEC culture conditions and stimulation

Mammary tissue samples were collected from healthy lactating cows of the INRA dairy facility (INRA, Nouzilly) at culling. Cows were slaughtered according to procedures approved by our Institutional Animal Care and Use Committee (CREEA, Comité Régional d’Ethique et d’Experimentation Animale). Bovine MEC were isolated from five cows as previously described and cryopreserved in liquid nitrogen [22]. When needed, the cells were thawed and cultured in RPMI 1640/DMEM (1:1) (Gibco) supplemented with 10% heat-treated FCS (Gibco), hydrocortisone (1 μg/mL), 40 mM HEPES (Cambrex Biowhittaker, East Rutherford, NJ, USA), insulin (5 μg/mL), 1 mM sodium pyruvate, 2 mM glutamin, 100 U penicillin, streptomycin (0.1 mg/mL), and amphotericin B (0.25 μg/mL). For the microarray experiment, the cells from one cow were seeded in 25 cm^2^ flasks (5 × 10^5^ cells/flask) and cultured until confluence in 5 mL of growth medium. The response of this cell batch in terms of CXCL8 production and TNF-α overexpression had proved to be representative of the other MEC prepared in our laboratory (results not shown). Cells were used at their third passage. Twenty-four hours before stimulation, the growth medium was replaced by a stimulation medium of the same composition except that the FCS concentration was lowered to 5% to reduce its influence on MEC response, and hydrocortisone was omitted. For stimulation of MEC with bacterial agonists, the medium was removed, and 20 μg/mL crude LPS (O55:B5) or 25% (v/v) SaS N305 in stimulation medium were added at concentrations that were supposed to induce robust responses on the part of MEC. After incubation for 3 h and 6 h, cell culture supernatant was removed and MEC were harvested for RNA extraction.

For confirmation of the results of microarray analysis by RT-qPCR, cells from 5 cows were seeded in 6-well tissue culture plates (2 × 10^5^ cells/well) and cultured until confluence. MEC were incubated with 20 μg/mL crude LPS or 25% SaS N305 in 2 mL of stimulation medium for 3 h and 6 h. At the end of incubation, the cell culture medium was aspirated and stored at -20°C and total RNA was extracted from the MEC layer.

To check whether the differential response of MEC to crude LPS compared to SaS involved the contribution of type I interferon, MEC were incubated with recombinant human IFN-β (produced in CHO cells; PeproTech, Rocky Hill, NJ, USA). The response of MEC to IFN-β was evaluated by RT-qPCR through the up-regulation of the IFN-inducible genes *Isg15* and *Ccxl10*, and appropriate concentration and exposure time were determined. Then, the effect of the exposure to 10 ng/mL IFN-β for 3 h before addition of SaS was determined by RT-qPCR after a further 3-h incubation.

### Assessment of viability of MEC

In the presence of 5% FCS, 8-h SaS N305 exerted moderate cytotoxic effect on bMEC after overnight incubation, whereas crude LPS did not cause any visual modification when compared to control culture conditions. The short term effect of LPS and SaS on the viability of MEC was evaluated using the alamarBlue® (Biosource International Camarillo, CA, USA) assay. MEC from 5 cows were cultured to confluence in 24-well culture plates and incubated for 3 h or 6 h with 10% alamarBlue in 1 mL of culture medium with or without 5% FCS. Fluorescence of the oxidation-reduction indicator was measured at 530 nm excitation wavelength and 590 emission wavelength (Cytofluor 2300 System). The results are expressed as means of the 5 cultures.

### Quantification of CXCL8, TNF-α and IL-1β

Enzyme-linked immunosorbent assays (ELISA) were used to measure cytokine concentrations in culture supernatants of MEC stimulated with LPS or SaS. The ELISA for bovine TNF-α and CXCL8 were performed as previously described [27,28]. Commercially available kits were used according to the manufacturer’s instructions to measure bovine IL-1β (Thermo scientific, Rockford, IL, USA) and human CXCL8 (Development kit, Peprotech). Quantifications of proteins by ELISA were performed on cell culture supernatants after 6 or 16 h of incubation with the agonists.

### RNA extraction and quality assessment

Total RNA was extracted by a double extraction method first using Trizol (InvitroGen) and then RNeasy (Qiagen, Valencia, CA, USA) column purification. RNA quantification was performed using a spectrophotometer (NanoDrop Technologies) and RNA integrity was assessed using an Agilent Bioanalyzer 2100 (Agilent Technologies, Inc., Santa Clara, CA, USA) with a RNA Integrity Number (RIN) value > 7.0. The residual genomic DNA was removed by DNA digestion with RNase-free DNase (Qiagen).

### Microarray experiment and analysis

Sixteen microarrays (2 stimuli * 2 times * quadruplicate) were hybridized using the ARK-Genomics 17 K slide (ArrayExpress accession number A-MEXP-1592) in a two-color dye swap experimental design (Additional file 2). RNA labeling was carried out with the fluorescent cyanine dyes Cy3 or Cy5 and hybridizations performed in a GeneTac automated hybridization station (Genomic Solutions) as described previously [29]. The data were extracted using BlueFuse [30]. Raw data were imported in R for filtering and normalization. Data were first filtered according to spot quality (uniformity and saturation) provided by BlueFuse. Spots with the worst quality (flag = E and quality = 0) were excluded, resulting in a removal rate of 33% to 45% per slide. Lowess normalization commonly used to correct the dye bias effect was not applied since MA-plots did not show any significant deviation from linearity. The log_2_-intensity of each dye was analyzed separately, since Bossers et al. [31] showed that this method enhances the reproducibility and the sensitivity of the detection of differentially expressed genes. Data were then reduced and centered within slide (mean intensity equal to zero and variance equal to one on average for the spots of one slide) to allow comparison across slides. Differences of expression between conditions were analyzed probe by probe by analysis of variance (ANOVA) using the SAS MIXED procedure. Probes with less than 16 observations were excluded from the analysis, resulting in an average of 28.5 observations per probe (± 9). Finally, 9559 probes and 272 770 observations (per dye) were retained. The ANOVA model included the fixed effect of color and the effect of the combination between stimulation (LPS, SaS and reference) and time (3 h, 6 h). The p-values of the tests were corrected with a 1% false discovery rate (FDR) with Benjamini-Hochberg correction [32]. Fold-changes (FC) were computed as the exponential (base 2) of the ANOVA-estimated-log_2_-intensity-ratio of samples from two conditions. The data discussed in this publication have been deposited in NCBI’s Gene Expression Omnibus and are accessible through GEO series accession number GSE47599.

To reveal functional connections between the regulated transcripts, a network and pathway analysis of the differentially expressed genes was performed by using Genomatix GePS (Genomatix Software GmbH, Munich, Germany). Lists of differentially expressed genes were input into the system with human ortholog gene names, along with their fold changes. Signal transduction pathways such as canonical and proprietary (Genomatix), molecular functions (Gene Ontology) and biological processes (GO) were listed provided the associated p value was > 10^-5^ (the threshold was lowered to 10^-4^ whenever none were > 10^-5^), and the number of genes in the list falling in the pathway > 5.

### Reverse transcription and PCR analysis (RT-qPCR)

From the samples used in the microarray analysis, total RNA was reverse transcribed to cDNA: 1 μg of RNA was incubated with 1 μg of random primers (Promega, Madison, WI, USA) for 10 min at 65°C and then for 5 min on ice in a final volume of 10 μL. Reverse transcription was carried out by adding avian myeloblastosis virus (AMV) reverse transcriptase buffer (Promega), 4 mM deoxynucleoside triphosphate (dNTP) (Promega), 15 U of AMV reverse transcriptase (Promega), and 40 U of RNasin (Promega) to the mixture. The mixture was incubated for 1.5 h at 42°C and 5 min at 95°C. Diluted cDNA samples were stored at 4°C until use. The same cDNA samples were used for microarray and qPCR determinations.

All primers (Table 2) used in this study were designed by using Clone Manager 9 (Scientific & Educational Software, Cary, NC, USA) using publicly available bovine sequences and were purchased from Eurogentec (Liège, Belgium). Primers were designed to span an intron-exon boundary to prevent the amplification of genomic DNA. Relative quantities of gene transcripts were measured by qPCR using the SYBR Green I fluorophore (Roche Diagnostics, Mannheim, Germany) as described previously [33].

**Table 2:** Gene-specific oligonucleotide primers used for qPCR

**Gene symbol**	**Oligonucleotides (5′-3′)**	**Amplicon**	**Annealing**	**Accession**
	**F: forward ; R: reverse**	**(bp)**	**Temperature (°C)**	**number (GenBank)**
18 s rRNA	F: CGGGGAGGTAGTGACGAAA	196	69	AF176811
	R: CCGCTCCCAAGATCCAACTA			
TNFa	F:TCTTCTCAAGCCTCAAGTAACAAGC	104	69	EU276079
	R: CCATGAGGGCATTGGCATAC			
Il6	F: TGCTGGTCTTCTGGAGTATC	153	62	EU276071
	R: GTGGCTGGAGTGGTTATTAG			
Ccl2	F: GCTCGCTCAGCCAGATGCAA	117	62	NM174006
	R: GGACACTTGCTGCTGGTGACTC			
Ccl5	F: CTGCCTTCGCTGTCCTCCTGATG	217	62	NM175827
	R: TTCTCTGGGTTGGCGCACACCTG			
Ccl20	F: TTCGACTGCTGTCTCCGATA	172	62	NM174263
	R: GCACAACTTGTTTCACCCACT			
CD83	F: GAA GGG CAG AGA AAC CTG AC	231	65°C	BC112861
	R : AGA GGT GAC TGG GAG GAA AG			
Nos2	F: CTT GAG CGA GTG GTG GAT GG	240	64	NM001076799
	R: CCT TCA TCC TGG ACG TGG TTC			
Isg15	F: CGC-CCA-GAA-GAT-CAA-TGT-GC			NM_174366
	R: TCC-TCA-CCA-GGA-TGG-AGA-TG	158	62	
Cxcl10	F: TTC-AGG-CAG-TCT-GAG-CCT-AC	218	62	NM_001046551
	R: ACG-TGG-GCA-GGA-TTG-ACT-TG			

### Statistical analyses

Statistical analyses were performed with the StatXact software (Cytel software Corp., Cambridge, MA, USA) or the Prism software (version 5.0; GraphPad). A probability level of < 0.05 was considered significant. Comparisons of paired samples were done with the Friedman test followed by Bonferroni post-test comparison and the comparison of unpaired samples with the Kruskal-Wallis test.

## Results

### Detection of PRR agonists in the LPS and SaS preparations

**Figure 1:**
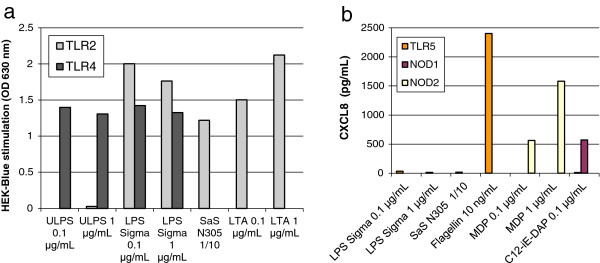
**Activity of crude LPS (LPS Sigma) and SaS (SaS N305) on HEK 293 cells transfected with human TLR2, TLR4, TLR5, Nod1 or Nod2. a**) Pure LPS (ULPS) and purified LTA were used as agonists of TLR4 and TLR2, respectively. The response of HEK Blue cells (InvivoGen) was measured spectrophotometrically (OD 630 nm). **b**) Flagellin, C12-iE-DAP and MDP were used as agonists of TLR5, Nod1 and Nod2, respectively. The response of HEK 293 cells was evaluated through secretion of CXCL8, measured by ELISA.

Because *E. coli* is sensed by sentinel cells of the immune system through activation of several PRR [34], we used a crude LPS preparation to stimulate bMEC. We investigated the presence of agonists of TLR2, TLR4, TLR5, Nod1, and Nod2 in the LPS preparation by incubating HEK293 cells transfected with the corresponding human PRR. We found that the crude LPS preparation activated HEK293 cells transfected with TLR2 and TLR4 (Figure 1a), but not TLR5, Nod1, and Nod2 (Figure 1b).

By using the same HEK 293 reporter cells, we found that 8-h SaS contained activators of TLR2 but not TLR4 or TLR5, and did not activate Nod1 or Nod2 (Figure 1a and b). As *S. aureus* N305 produces several toxins, including alpha hemolysin which is known to be cytotoxic, we determined whether SaS altered the metabolism of MEC under our culture conditions by using an indicator of chemical reduction of growth medium (alamarBlue). Neither 25% (v/v) 8-h SaS nor LPS (20 μg/mL) induced a significant effect on the activity of MEC after 3 h and 6 h of incubation, indicating that MEC were neither impaired nor activated (Additional file 3).

**Figure 2:**
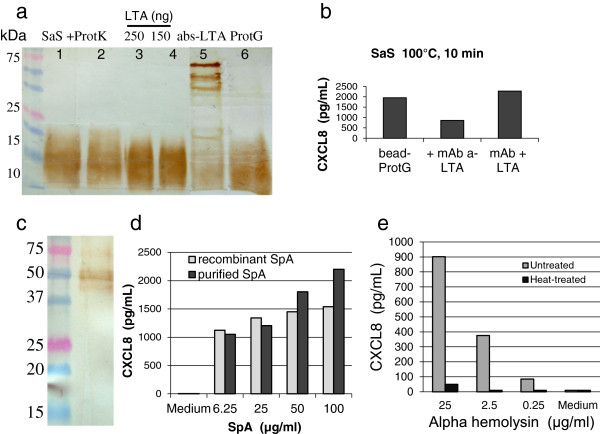
**Detection of MAMP in SaS and effect on MEC. a**) Detection of LTA in SaS. SaS (50 μL) was submitted to SDS-PAGE and immunoblotted with a mAb to LTA (track 1). LTA appeared as a smear owing to its size heterogeneity as a result of varied number of glycerolphosphate units. Immunoblotting was also performed with SaS treated with proteinase K (track 2) or with purified *S. aureus* LTA (250 ng or 150 ng, tracks 3 and 4). SaS was depleted of LTA with beads coated with mAb to LTA (track 5). High molecular weight bands are the mAb light and heavy chains. SaS treated with control beads without mAb is shown (track 6). **b**) MEC were incubated for 16 h with SaS (25%) treated with control beads or with mAb-coated beads, and concentrations of CXCL8 in cell culture supernatants measured by ELISA. Purified LTA (250 ng/mL) was added to the depleted SaS to restore activity. **c**) Staphylococcal protein A (SpA) was detected in SaS and was able to stimulate bMEC. Immunoblot of 8-h N305 SaS revealed with a peroxidase-conjugated rabbit antiserum reacting with SpA; **d**) bMEC were incubated for 6 h with either recombinant SpA or purified SpA, and CXCL8 concentrations measured in the cell culture supernatant by ELISA. **e**) Dose–response of bMEC to decreasing concentrations of staphylococcal alpha hemolysin. Bovine MEC were incubated with purified alpha hemolysin for 16 h, and CXCL8 was measured in cell culture supernatant by ELISA. To investigate the heat-resistance of the stimulus, alpha hemolysin was heated at 95°C for 10 min.

Anticipating that SaS contains multiple bacterial components including exoproteins, lipoproteins and PGN derivatives that are susceptible to stimulate MEC, we verified the presence of some of these components and determined their capacity to activate MEC. We used the secretion of CXCL8 as a readout for MEC stimulation experiments. Since the response of HEK293 -TLR2 cells indicated that SaS contains at least one agonist of TLR2, we looked for the presence of LTA, which is supposed to be sensed by TLR2 in association with CD36 [35] and is able to activate bMEC [10,33]. SaS was submitted to SDS-PAGE and immunoblotted with a mAb against LTA, along with purified staphylococcal LTA. The immunoblotting revealed a smear comparable to that of purified LTA, which by comparison with known LTA concentration was estimated to be 1 to 2 μg/mL (Figure 2a). Depletion experiments were carried out to investigate whether LTA contributed to the response of MEC to SaS. Incubation of SaS with beads coated with the anti-LTA mAb was efficient in depleting SaS of LTA, and this depletion correlated with a reduced stimulating activity of SaS on bMEC (Figure 2a and b). This showed that LTA is likely to contribute to the activation of MEC, but suggests that other staphylococcal products play a part in the stimulation.

Since staphylococcal protein A (SpA) is able to induce a pro-inflammatory response in human epithelial cells [36], we looked for the presence of SpA in SaS. SDS-PAGE followed by immunoblotting revealed the presence of SpA in 8-h SaS (Figure 2c). We then investigated whether bMEC responded to SpA, because we did not find previously published evidence of recognition of SpA by these cells. Incubation of bMEC with increasing concentrations of either recombinant or native purified SpA induced increasing secretion of CXCL8, indicating that bMEC reacted to SpA (Figure 2d).

It has been shown that epithelial cells detect the presence of bacterial pore-forming toxins such as staphylococcal hemolysin alpha [37]. As *S. aureus* N305 produces high amounts of α-hemolysin and as we did not find published evidence that *S. aureus* α-hemolysin induced a pro-inflammatory response in bMEC on its own, we investigated the capacity of purified α-hemolysin to induce the secretion of CXCL8 by MEC. We found that bMEC responded to α-hemolysin by secreting CXCL8 in a dose–response manner (Figure 2e). This activity was heat-labile, which suggests that it was not the result of the contamination of the toxin preparation with MAMP (Figure 2e).

Overall, these experiments indicate that N305 SaS contains several bacterial compounds that are able to stimulate bMEC, and identified LTA, SpA and α-hemolysin, as three of its active components which interact with different sensors which activate downstream pathways.

### Transcriptome profiling of the response of bMEC to LPS preparation and SaS

The reaction of MEC to LPS and SaS was investigated through gene expression profiling at a rather early time post-exposure in order to gain insight into the response of the cells likely to contribute to the triggering of the inflammatory response.

**Figure 3:**
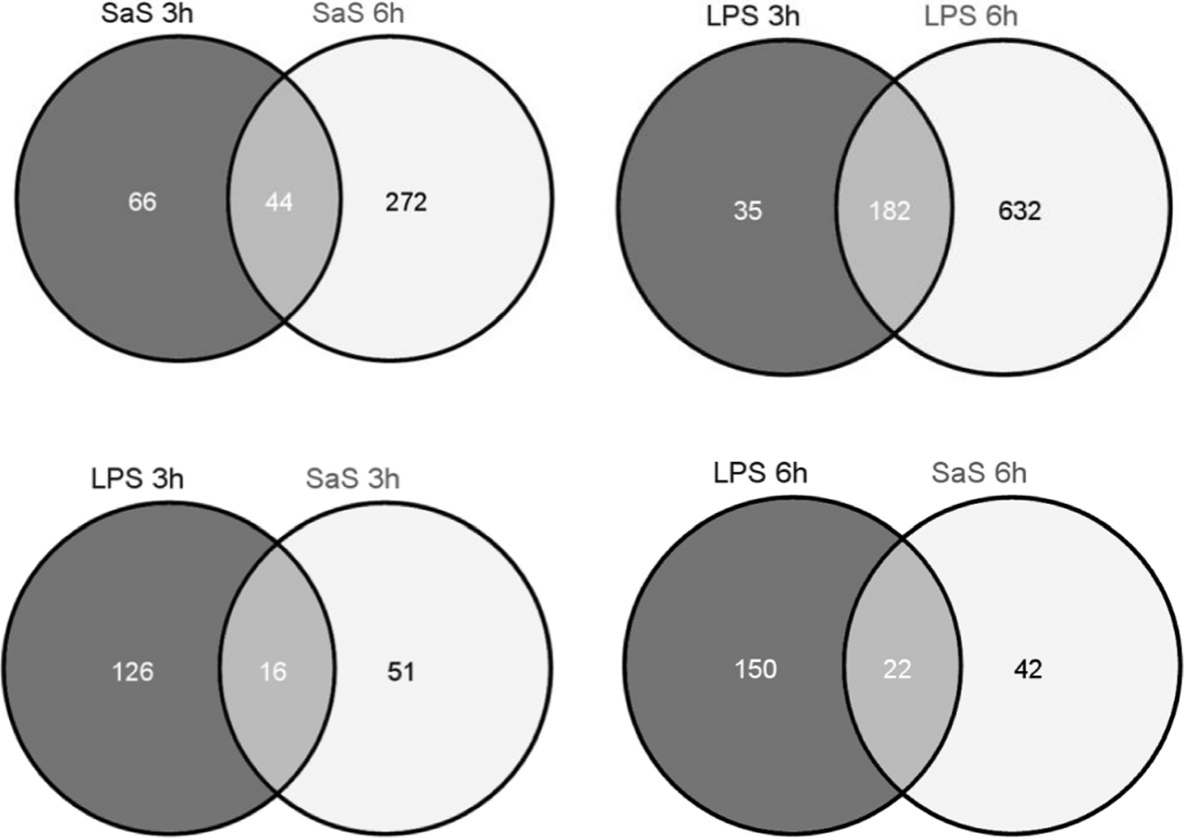
**Venn diagrams showing differentially expressed genes as a function of time and stimulus.** Upper row: all DEG. Lower row: up-regulated annotated genes only. The lists of the sets of genes up-regulated on exposure to both SaS and LPS are given. Venn diagrams composed with VENNY [38]. List of the 16 upregulated annotated genes shared in LPS 3 h and SaS 3 h: CCL20, CXCL8, IL6, CD83, CXCL1, NFKBIA, CXCL3, BIRC3, SLCO4A1, PLAUR, IER3, ARRDC4, CEBPD, SGK1, BTG3, GABARAPL1. List of the 22 upregulated annotated genes shared in LPS 6 h and SaS 6 h: CCL20, CXCL8, CXCL5, NOS2A, CCL2, CXCL2, CFB, SGK1, NFKBIA, CD83, ARRDC4, SLC25A28, SAT1, IKBKAP, GABARAPL1, CALCOCO2, PLAUR, LEPROT, DUSP1, TLR4, CEBPD, SNRNP27.

Data were validated for 9559 probes on the basis of the Reml with a Benjamini-Hochberg correction of 1%. Microarray analysis of bMEC RNA obtained 3 h and 6 h post-exposure to LPS or SaS revealed differential expression of hundreds of genes by comparison with unstimulated cells (Additional file 4). More genes were differentially expressed at 6 h than at 3 h post-exposure, and following exposure to LPS than to SaS (Table 3). Also, the proportion of down-regulated genes was higher with SaS than with LPS at 6 h post-exposure (Table 3). There was a marked time effect on the expression of genes because only 44 out of 382 and 182 out of 849 differentially expressed genes (DEG) belonged to the sets of genes expressed 3 h and 6 h after exposure to SaS or LPS, respectively (Figure 3). Most of the up-regulated genes differed also as a function of the stimulus at both 3 h and 6 h post-exposure (Figure 3). The shared up-regulated genes coded mainly for chemokines, cytokines, or molecules associated with the inflammatory and immune response (Figure 3).

**Table 3:** Number of differentially expressed genes (DEG) as a function of stimulus and duration of exposure

**DEG**	**Sas vs Ref 3 h**	**SaS vs Ref 6 h**	**LPS vs Ref 3 h**	**LPS vs Ref 6 h**
Up-regulated	103	104	201	541
Down-regulated	7	212	16	273

On the basis of annotation to human or mouse orthologs and information obtained using NCBI and InnateDB [39], the most differentially expressed genes were distributed in categories related to different biological processes (Tables 4 and 5). From the lists of the most highly up-regulated genes at 3 h and 6 h of incubation with either LPS or SaS, a few indications can be drawn: i) Fold changes were higher with LPS than with SaS, which reflects a stronger response to LPS than to SaS; ii) chemokines were highly represented among the most up-regulated genes. Chemokines with the ELR motif preceding the first two cysteines (ELRCXC chemokines), which target mainly neutrophils, were strongly up-regulated (CXCL1, CXCL2, CXCL3, CXCL8). Notably, several chemokines which target monocytes and lymphocytes, namely CCL2, CCL5 and CCL20, were also up-regulated. iii) A distinctive feature of the response of bMEC to LPS was the involvement of IFN-related genes and IFN-β itself, which were not up-regulated by SaS. iv) a characteristic of the bMEC response to SaS was the up-regulation of genes implicated in the regulation of transcription and activation pathways, and in particular the NF-κB pathway, among the most up-regulated genes (Table 4). A further difference between responses to LPS and SaS was the greater occurrence of down-regulated genes in response to SaS (Table 4). Another difference was that genes encoding proteins of the activator protein-1 (AP-1) complex were up-regulated by SaS, but not by LPS. In contrast, *Fos* was down-regulated at 6 h by LPS.

**Table 4:** Genes most significantly differentially expressed by bMEC stimulated by LPS for 3 and 6 h compared to unstimulated cells, ordered by functional classes

**Gene symbol**	**Gene description**	**Fold change**	
		**3 h**	**6 h**
***Transcription and activation pathways***			
NFKBIA	Nuclear factor of kappa light polypeptide gene enhancer in B-cells inhibitor, alpha	3.60	4.53
BIRC3	Baculoviral IAP repeat-containing protein 3	3.10	3.19
ZNFX1	NFX1-type zinc finger-containing protein 1		3.11
ZNHIT3	Zinc finger HIT domain-containing protein 3	2.48	3.08
IKBKAP	Inhibitor of kappa light polypeptide gene enhancer in B-cells, kinase complex-associated protein	2.52	2.33
TRIM21	Tripartite motif-containing protein 21	1.61	2.26
CEBPD	CCAAT/enhancer binding protein (C/EBP), delta	2.02	1.60
NFKB2	Nuclear factor of κ light polypeptide gene enhancer in B-cells 2	1.78	1.94
JAK2	Janus kinase 2	1.90	1.85
DUSP1	Dual specificity phosphatase 1		1.85
FOS	V-fos		-2.01
PLK2	Serum-inducible kinase, transcript variant 1,PLK2	-1.50	
***Cytokines and chemokines, growth factors***			
CCL5	RANTES	7.10	20.52
CCL20	Chemokine (C-C motif) ligand 20	19.42	19.37
CXCL8	Chemokine (C-X-C motif) ligand 8, Interleukin-8	8.53	13.96
CXCL5	Chemokine (C-X-C motif) ligand	4.75	7.96
CCL2	Chemokine (C-C motif) ligand 2		6.38
CXCL2	Chemokine (C-X-C motif) ligand 2		5.06
CXCL1	Chemokine (C-X-C motif) ligand 1	4.93	
IL6	Interleukin 6	6.64	4.85
CXCL3	Chemokine (C-X-C motif) ligand 3	3.15	3.60
IL1B	Interleukin 1, beta	5.01	3.32
IFNB	Interferon beta precursor	4.89	3.25
TNFSF13B	Tumor necrosis factor (ligand) superfamily, member 13 (BAFF)	2.96	
IL2	Interleukin 2	4.30	2.86
CCL16	Chemokine (C-C motif) ligand 16	2.60	2.77
CSF2	Colony stimulating factor 2 (granulocyte-macrophage)	1.69	2.10
IL23A	Interleukin 23, alpha subunit p19		2.06
CTGF	Connective tissue growth factor	-1.92	
TGFB2	Transforming growth factor beta-2 precursor		-1.46
***Type I IFN-related genes***			
IFIT3	Interferon-induced protein with tetratricopeptide repeats 3	3.02	7.92
MX1	Myxovirus (influenza virus) resistance 1	1.84	2.93
IFIH1	Interferon induced with helicase C domain 1	2.65	4.10
ISG15	ubiquitin-like modifier	2.46	3.69
GBP1	Interferon-induced guanylate-binding protein 1	2.17	3.56
TNFSF10	Tumor necrosis factor ligand superfamily member 10 (TRAIL)	2.02	3.13
IFI44	Similar to Interferon-induced protein 44		3.23
OAS1	2′-5′-oligoadenylate synthetase 1	1.61	3.15
IFI27L1	Interferon alpha-inducible protein 27-like protein 1		2.88
IRF7	Interferon regulatory factor 7	1.45	2.75
TRIM21	Tripartite motif-containing protein 21	1.61	2.26
GVIN1	GTPase, very large interferon inducible 1		2.21
STAT2	Signal transducer and activator of transcription 2		2.01
STAT1	Signal transducer and activator of transcription 1		1.96
IRF2	Interferon regulatory factor 2		1.84
***Immune defense response***			
NOS2A	Nitric oxide synthase 2A (inducible)	5.57	7.45
S100A8	Calcium-binding protein A8, calgranulin A	2.87	7.00
CFB	Complement factor B	3.39	5.02
S100A9	Calcium-binding protein A9, calgranulin B	2.40	4.59
CD83	Cell surface protein HB15	5.50	3.40
C2	C2 Complement component 2	1.79	2.64
ICAM1	Intercellular adhesion molecule 1	2.50	
LTF	Lactotransferrin		2.47
PLAT	Plasminogen activator, tissue type	1.93	2.11
PLAUR	Urokinase plasminogen activator surface receptor	2.11	1.96
CAMP	Cathelicidin antimicrobial peptide		1.89
TLR4	Toll-like receptor 4	1.62	1.81
MMP9	Matrix metallopeptidase 9		1.77
CD74	Major histocompatibility complex, class II invariant chain		1.75
BNBD10	Beta-defensin 10	1.59	1.67
***Stress response***			
SOD2	Superoxide dismutase 2	3.19	5.05
SGK1	Serum/glucocorticoid regulated kinase 1	1.81	4.79
PARP14	Poly (ADP-ribose) polymerase family, member 14		2.95
***Cell growth and cycle, apoptosis***			
IGFBP3	Insulin-like growth factor binding protein-3	2.38	5.27
EIF4E	Translation initiation factor 4E	2.32	5.20
CASP4	Caspase-4 Precursor	1.60	3.04
CASP8	Caspase-8 precursor	1.58	2.29
IER3	Immediate early response 3	2.09	
TNFRSF6	TNF receptor superfamily, member 6 (FAS)	1.82	1.86
CDK2AP1	Cyclin-dependent kinase 2 associated protein 1		-1.66
EI24	Etoposide-induced protein 2.4 homolog		-1.65

**Table 5:** Genes most significantly differentially expressed by bMEC stimulated by SaS for 3 and 6 h compared to unstimulated cells, ordered by functional classes

**Gene symbol**	**Gene description**	**Fold change**	
		**3 h**	**6 h**
***Transcription and activation pathways***			
FOS	Proto-oncogene protein c-fos	2.09	2.21
NFKBIA	Nuclear factor of kappa light polypeptide gene enhancer in B-cells inhibitor, alpha	2.15	2.07
DUSP1	dual specificity phosphatase 1	1.91	1.71
ETS2	erythroblastosis virus E26 oncogene homolog 2 (avian)		1.72
CEBPD	CCAAT/enhancer binding protein (C/EBP), delta	1.65	1.59
SQSTM1	sequestosome 1		1.58
BIRC3	Baculoviral IAP repeat-containing 3	1.56	
IKBKAP	inhibitor of kappa light polypeptide gene enhancer in B-cells, kinase complex-associated protein		1.49
JUN	Jun oncogene	1.57	1.47
NFKB2	nuclear factor of κ light polypeptide gene enhancer in B-cells 2	-2.05	
FUS	RNA-binding protein FUS		-1.82
SFRS3	Splicing factor, arginine/serine-rich 3		-1.71
SFPQ	splicing factor proline/glutamine-rich		-1.59
RABL3	RAB, member of RAS oncogene family-like 3		-1.57
U2AF1	Splicing factor U2AF		-1.54
CSTF2	cleavage stimulation factor, 3′pre-RNA, subunit 2		-1.53
RDBP	RNA-binding protein RD, Negative elongation factor E		-1.47
DKC1	dyskeratosis congenita 1, dyskerin		-1.47
CRSP9	Cofactor required for Sp1 transcriptional activation subunit 9	-1.45	
***Cytokines and chemokines***			
CXCL8	Chemokine (C-X-C motif) ligand 8, Interleukin-8	6.20	4.52
CCL20	chemokine (C-C motif) ligand 20	4.94	4.27
CXCL1	Chemokine (C-X-C motif) ligand 1	3.51	
CXCL3	Chemokine (C-X-C motif) ligand 3	2.48	
CCL2	chemokine (C-C motif) ligand 2	2.22	2.71
CXCL5	chemokine (C-X-C motif) ligand 5		2.57
CXCL2	chemokine (C-X-C motif) ligand 2		2.47
IL6	Interleukin 6	1.71	
VEGFB	Vascular endothelial growth factor B Precursor		1.47
***Immune defense response***			
PLAUR	urokinase plasminogen activator surface receptor	1.70	2.46
LBP	lipopolysaccharide binding protein		2.26
NOS2A	Nitric oxide synthase 2A (inducible)		2.05
CD83	Cell surface protein HB15	1.89	1.73
CFB	complement factor B		1.70
SQSTM1	sequestosome 1		1.58
TLR4	toll-like receptor 4		1.50
ILRAP	interleukin 1 receptor accessory protein		1.44
CD96	Cell surface antigen CD96		1.31
C3	Complement factor 3		-1.35
MMP9	Matrix metallopeptidase 9	-1.33	
***Stress response***			
SGK1	serum/glucocorticoid regulated kinase 1	1.73	1.75
***Cell growth and cycle, apoptosis***			
IER3	immediate early response 3	1.99	1.58
RASSF1	Ras association domain-containing protein 1		1.52
CDKN2B	cyclin-dependent kinase inhibitor 2B		-1.85
CDC20	cell division cycle 20 homolog (S. cerevisiae)		-1.60
CCNA2	cyclin A2		-1.37

In the cytokine and chemokine category, more genes were up-regulated by LPS than by SaS. The difference was most striking in the cytokine category, which was represented only by *IL6* with SaS stimulation. The case of IFN-β is of particular interest, because a number of genes related to the type I IFN-pathway were upregulated following exposure to LPS, but not to SaS.

A number of genes associated with the immune defense response, and in particular with innate immunity, were up-regulated by both LPS and SaS. Again, more genes were up-regulated after exposure of bMEC to LPS than to SaS. Genes associated with cell growth, cell cycle, apoptosis or stress response were also involved in the response to LPS or SaS (Tables 4 and 5).

We had anticipated that the response of bMEC would differ substantially with the duration of incubation with bacterial agonists. Indeed, higher numbers of genes were differentially regulated with the 6-h incubation than with the 3-h incubation, with the highest differences for up-regulated genes for LPS and down-regulated genes for SaS (Table 2). On the contrary, many of the genes most regulated by exposure to LPS at 3-h were also highly regulated at 6-h (Table 4). This was not true after exposure to SaS, because a substantial proportion of the differentially expressed genes (DEG) at 3-h differed from those at 6-h (Table 5). After exposure to both LPS and SaS, genes coding transcription factors, cytokines or chemokines tended to be more up-regulated at 3-h than at 6-h, whereas genes associated with immune defense response, type I IFN (for LPS), stress response and cell cycle tended to be more up-regulated at 6-h than at 3-h (Tables 4 and 5). Overall, this suggests that the activation pathways and the early chemokine and cytokine production preceded the defense and stress responses, which was not unexpected.

A functional analysis of the genes up-regulated after stimulation was performed with Genomatix GePS to display canonical pathways and to create networks. In response to LPS, most of the highest ranking signal transduction pathways, molecular functions and biological processes were connected to inflammatory and immune responses (Table 6). In contrast, the signal transduction pathways associated with the response of MEC to SaS at 6 h post-exposure were connected with cell cycle and DNA repair (Table 7). Nevertheless, at 3 h post-exposure to SaS, the AP-1 transcription factor network was identified, along with the pro-inflammatory IL-17A pathway (Table 7). This analysis confirmed the distinct nature of the responses of MEC to the two stimuli.

**Table 6:** Functional pathways of genes most affected by LPS stimulation, determined by using the Genomatix Pathway System (GePS)

**LPS vs Ref 3 h (List of 147 genes taken into account)**		
	***p *value**	nb of genes*
**Signal transduction pathways (canonical)**		
Fas signaling pathway	7.58 × 10^-6^	5/20
**Signal transduction pathways (Genomatix)**	*p* value	nb of genes
NF kappa B	1.52 × 10^-12^	41/1542
Signal transducer and activator of transcription	1.76 × 10^-12^	36/1184
Toll like receptor	2.49 × 10^-12^	26/593
Apoptosis	7.27 × 10^-12^	52/2592
Tumor protein p53	8.23 × 10^-12^	34/1108
Interleukin 1	2.05 × 10^-11^	27/706
Janus kinase	2.57 × 10^-11^	26/657
Tumor necrosis factor	5.10 × 10^-11^	34/1182
Interleukin 6	5.57 × 10^-11^	26/680
Inflammatory	8.31 × 10^-11^	33/1133
Nuclotide oligodimerization domain/	9.52 × 10^-11^	13/131
Caspase recruitment protein family		
**Molecular functions (GO)**		
Cytokine receptor binding	2.29 × 10^-9^	12/187
Receptor binding	9.83 × 10^-9^	23/917
Cytokine activity	5.22 × 10^-8^	11/199
Chemokine activity	4.88 × 10^-7^	6/47
Chemokine receptor binding	9.02 × 10^-7^	6/52
**Biological processes (GO)**		
Immune system process	2.27 × 10^-11^	28/1094
Immune response	2.98 × 10^-10^	22/736
Locomotion	2.72 × 10^-9^	19/605
Multi-organism process	8.37 × 10^-9^	21/800
Response to chemical stimulus	1.67 × 10^-8^	28/1461
**LPS vs Ref 6 h** (List of 193 genes taken into account)		
**Signal transduction pathways (Genomatix)**		
Signal transducer and activation	8.41 × 10^-17^	44/1184
NF-kB	2.66 × 10^-16^	49/1542
Chemokine (CC motif) ligand 2	8.63 × 10^-16^	23/272
Interleukin 6	1.91 × 10^-15^	33/680
Toll like receptor	2.39 × 10^-15^	31/593
Immune	4.25 × 10^-15^	38/957
Inflammatory	4.85 × 10^-15^	41/1133
Tumor necrosis factor	2.14 × 10^-14^	41/1182
Interleukin 1	2.87 × 10^-13^	31/706
Janus kinase	2.89 × 10^-13^	30/657
**Molecular functions (GO)**		
Cytokine activity	1.21 × 10^-10^	14/199
Receptor binding	3.74 × 10^-9^	25/917
Cytokine receptor binding	7.90 × 10^-9^	12/187
Protein binding	5.75 × 10^-6^	80/8067
**Biological processes (GO)**		
Immune response	1.59 × 10^-14^	29/736
Immune system process	3.15 × 10^-13^	33/1094
Response to stress	1.53 × 10^-12^	42/1880
Defense response	2.45 × 10^-12^	26/708
Response to stimulus	2.98 × 10^-11^	58/3713

* Nb of genes: number of genes in the list/number of genes in the pathway.

**Table 7:** Functional pathways of genes most affected by SaS stimulation, determined by using the Genomatix Pathway System (GePS)

**SaS vs Ref 3 h (List of 65 genes taken into account)**		
	***p *value**	**nb of genes**
**Signal transduction pathways (canonical)**		
AP-1 transcription factor network	5.05 × 10^-7^	6/69
**Signal transduction pathways (Genomatix)**		
Interleukin 17A	8.75 × 10^-7^	7/137
Protein kinase B	1.59 × 10^-6^	9/297
Baculoviral IAP repeat containing protein,	1.94 × 10^-6^	9/304
apoptosis inhibitor		
FBJ murine osteosarcoma viral oncogene	6.49 × 10^-6^	4/32
homolog B		
Cyclin D1	7.69 × 10^-6^	10/461
Chemokine (CC motif) ligand 2	8.40 × 10^-6^	8/272
**Molecular functions (GO)**		
Too few genes, *p* value > 10^-5^		
**Biological processes (GO)**		
Too few genes, *p* value > 10^-5^		
**SaS vs Ref 6 h** (213 genes taken into account)		
**Signal transduction pathways (canonical)**		
Too few genes, *p* value > 10^-5^		
**Signal transduction pathways (Genomatix)**		
Minichromosome maintenance complex	6.13 × 10^-7^	7/42
Cyclin B1	9.29 × 10^-7^	10/112
Cyclin D1	2.34 × 10^-6^	19/461
DNA repair	4.43 × 10^-6^	23/725
Cyclin A2	4.37 × 10^-6^	11/167
Checkpoint	2.51 × 10^-5^	16/402
Interleukin 17A	4.07 × 10^-5^	10/137
Nuclear receptor subfamily 3, group C	4.08 × 10^-5^	10/171
Cell cycle	4.94 × 10^-5^	32/1309
Aryl hydrocarbon receptor	9.93 × 10^-5^	10/190
**Molecular functions (GO)**		
Protein binding	2.95 × 10^-5^	100/8067
Protein heterodimerizaton activity	1.23 × 10^-4^	9/202
Oxidoreductase activity	2.39 × 10^-4^	17/685
**Biological processes (GO)**		
Symbiosis, encompassing mutualism	2.15 × 10^-5^	6/58
Viral reproduction	3.84 × 10^-5^	7/94
Cell death	1.05 × 10^-4^	27/1275
Death	1.10 × 10^-4^	27/1279
Response to organic substance	1.24 × 10^-4^	21/874
Oxidation reduction	1.98 × 10^-4^	17/645

### Validation of microarray results and quantification by RT-qPCR or ELISA

**Figure 4:**
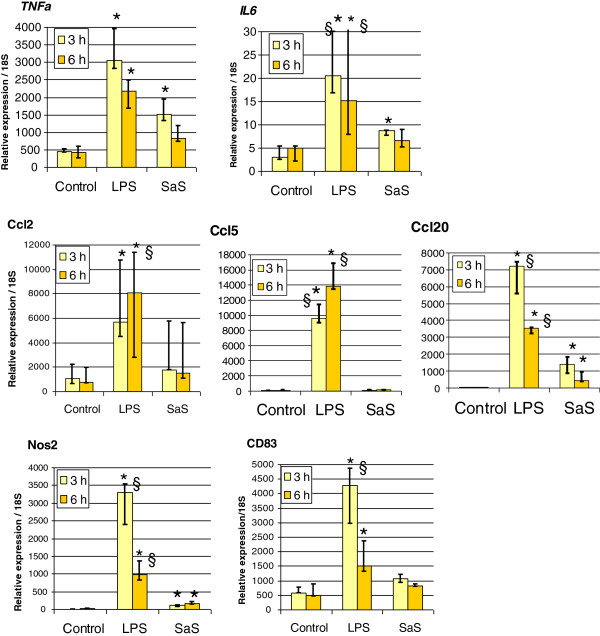
**Analysis by RT-qPCR of the expression of a set of genes showing differential expression in the microarray analysis.** Relative quantification of the *TNFa*, *IL6*, *Ccl2*, *Ccl5*, *Ccl20*, *Nos2* and *CD83* transcripts by RT-qPCR 3 h or 6 h after stimulation of bMEpC with LPS or SaS. Median values (Q1 and Q3) from bMEpC of five cows are shown. * Statistical significance relative to Control. § Statistical significance, LPS *versus* SaS.

RT-qPCR or ELISA validation confirmed the microarray results. Preliminary experiments indicate that TNF-α transcript levels increased following stimulation of bMEC with LPS and SaS (results not shown). Up-regulation of the gene encoding TNF-α (*TNFa* or *TNFSF2*) was not detected in the microarray analysis, because the number of validated observations was insufficient (< 16 validated spots). Quantification by RT-qPCR of the expression of *TNFa* shows that it increased significantly at 3 h and 6 h post-exposure to LPS and at 3 h post-exposure to SaS (Figure 4). Expression tended to be higher at 3 h than at 6 h, and with LPS than with SaS. The same pattern was obtained with *IL6* transcripts (Figure 4). It is noteworthy that the cytokines TNF-α and IL-1β were not detectable by ELISA in the supernatants of bMEC stimulated with LPS or with SaS.

Stimulated bMEC secreted CXCL8, with LPS inducing a higher response (median value 7846 pg/mL; range 3915 – 9323 pg/mL), than SaS (median 1721; range 1181 – 3090 pg/mL) (*p* < 0.01, *n* = 7, Wilcoxon test), after 8 h of incubation with the agonists, again indicating that LPS was a stronger inflammatory stimulus than SaS. Transcripts of 3 other chemokines were quantified by RT-qPCR, including CCL2 and CCL20 which were among the most up-regulated in the microarray analysis (Figure 4 and Tables 4 and 5) following stimulation with LPS and SaS. The results of RT-qPCR confirmed this observation, showing that the response was significantly higher with LPS than with SaS (Figure 4). Although *Ccl2* up-regulation had not been detected in the microarray analysis following 3-h exposure to LPS, it was by RT-qPCR. Also, increases in *Ccl2* expression following exposure to SaS were of limited magnitude and not statistically significant. The chemokine CCL5 (also known as RANTES) appeared in the list of genes up-regulated by LPS but not by SaS (Tables 4 and 5). This result was confirmed by RT-qPCR, which showed a marked upregulation of *CCL5* transcription with LPS but not with SaS (Figure 4).

The expression of *Nos2a*, a gene associated with the innate immune response encoding the inducible nitric oxide synthase, was up-regulated by LPS and SaS in the microarray analysis (Tables 4 and 5). Quantification by RT-qPCR confirmed the increases in transcript numbers and showed that LPS induced a significantly higher increase (*p* < 0.05) at both 3 h and 6 h post-exposure than did SaS (Figure 4). Since the up-regulation of *CD83* (Tables 4 and 5) was somewhat unexpected as this gene is mainly expressed by antigen-presenting cells [40], its expression was checked by RT-qPCR. The results indicate that transcript numbers were significantly increased only by LPS, at both 3 h and 6 h post-exposure (Figure 4).

### Putative contribution of type I IFN to the LPS-induced response of bMEC

The increased expression of type I IFN-related genes by LPS most likely resulted from the activation of the MyD88-independent IFN regulatory factor 3 (IRF3) signaling pathway of TLR4. This pathway leads to the nuclear translocation of IRF3 which binds to IRF-binding sites in the regulatory region of a number of so-called IFN-regulated genes, although IFN is not involved at this primary stage. Among the primary response genes is the gene encoding IFN-β, present as multiple copies in *Bos Taurus*[41], which creates a positive autocrine/paracrine feedback loop through the secretion of IFN-β, engagement of the IFN type I receptor (IFNAR) and binding of transcription factors to IFN-stimulated response elements (ISRE) or more generally IFN regulatory factor binding sites (IRFBS) [42]. As a result, secondary response genes are overexpressed [43]. We aimed to check whether bMEC respond to type I IFN by overexpressing IFN-related primary and secondary response genes.

**Figure 5:**
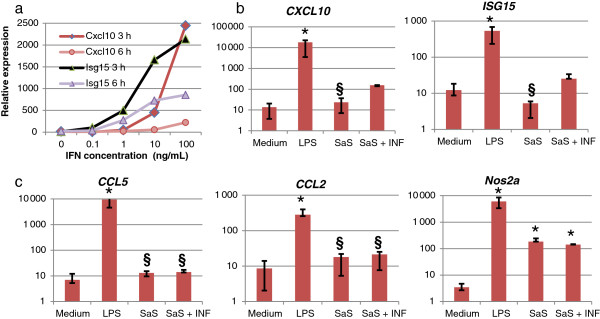
**Effect of IFN-β alone or in combination with SaS on the expression of IFN-inducible genes by bMEC. a**) Response of bMEC to increasing concentrations of recombinant human IFN-β. After 3 or 6 h of incubation with IFN-β, the relative expression of the *Cxcl10* and *Isg15* genes was determined by RT-qPCR. **b**) Effect of adding IFN-β (10 ng/mL) to SaS after 3 h of exposure to SaS, and comparison with the effect on *Cxcl10* and *Isg15* expression of SaS and LPs alone, measured 3 h after addition of IFN-β. **c**) Effect of adding IFN-β as in b) on the expression of *Ccl5*, *Ccl2* and *Nos2a*. * *P* < 0.05, relative to Medium; $ *P* < 0.05, LPS versus SaS.

Preliminary experiments were carried out to determine the working concentration of IFN-β, genes suitable as readouts, and the window time of response. Among several sources of ovine, bovine and human type I IFN, recombinant human IFN-β was the most active on bMEC (results not shown). The IFN-inducible genes *Isg15* and *Cxcl10* proved to be appropriate readouts for response to IFN-β yielding marked increased expression at 3 h of incubation with 10 ng/mL IFN-β or more (Figure 5a). The response to IFN-β decreased by 6 h of incubation (Figure 5a). Subsequently, the concentration of 10 ng/mL IFN-β was preferred because it is more in the physiological range. To mimic the effect of a postulated secretion of type I IFN in response to LPS stimulation, IFN-β (10 ng/mL) was added to MEC after 3 h of incubation with SaS, and the cells were incubated for a further 3 h. Control cells were incubated with medium alone or with medium plus LPS. Incubation with LPS induced an overexpression of *Cxcl10* and *Isg15*, whereas incubation with SaS did not (Figure 5b). The addition of IFN-β during the incubation increased the expression of these two genes 5 to 6 fold (Figure 5b), indicating that IFN-β was active under these conditions. Nevertheless, the expression of *Ccl5*, *Ccl2* and *Nos2a* was not increased by the addition of IFN-β to SaS (Figure 5c). This suggests either that the expression of these genes is less dependent on type I IFN than are *Cxcl10* or *Isg15*, or that SaS inhibited the effect of IFN-β on bMEC. It also suggests that the overexpression of *Ccl2*, *Ccl5* and *Nos2a* induced by LPS did not depend on an induced secretion of type I IFN, but rather that these genes behaved as primary response genes, presumably responding to LPS stimulation through the TLR4-related IRF3 pathway.

## Discussion

Mastitis caused by *E. coli* is usually clinical and short-lived due to self-cure whereas *S. aureus* commonly elicits mild clinical chronic mastitis. We and others are aiming to improve our knowledge of mammary gland immunity by contrasting the response of the mammary gland or mammary cells to these two pathogens [5,6,10,13,14,22,44,45]. In this study, we compared the response of bMEC to bacterial products released by multiplying *E. coli* or *S. aureus*.

The mastitis-causing pathogens *E. coli* and *S. aureus* which have penetrated into the lumen of the mammary gland are able to grow and multiply in milk with comparable generation time and concentration plateau [46]. Consequently the difference in severity and outcome of infections depends at least in part on the way the two pathogens are perceived and dealt with by the mammary gland. Following experimental infusion of small numbers of bacteria through the teat canal, several hours elapse before the first signs of inflammation manifest themselves [47]. During this lag phase, bacteria are likely to produce immunomodulins such as MAMP and virulence factors. We decided to investigate the response of the most abundant sentry cells in the mammary gland, i.e. MEC, to bacterial compounds representative of these released molecules. A crude LPS preparation was chosen as *E. coli* stimulus, and an early culture supernatant as *S. aureus* stimulus. Since we were interested in the early response of bMEC to *S. aureus*, we speculated that the supernatant from exponential or post-exponential culture phase would be more representative of the initial phase of infection rather than the supernatant of the stationary phase. Among MAMP detected in these stimuli, TLR4 and TLR2 agonists were present in the crude LPS preparation, and TLR2 agonists in the SaS. We identified LTA as one potential TLR2 agonist, but it is likely that lipoproteins were also major agonists in SaS [48]. Staphylococci are known to produce a number of agonists of the innate immune system [18]. We investigated the presence and potential activity of two of them, SpA and α-hemolysin. These two virulence factors were present in the 8-h SaS, and both were sensed by bMEC as shown by induction of CXCL8 secretion (Figure 2). Although these responses were not documented with MEC before, they are not unexpected. For example, SpA has been shown to induce CXCL8 production by activating TNFR1 signaling [49], and bMEC respond to TNF-α [50,51]. Alpha-hemolysin has been reported to induce CXCL8 in bMEC when used in association with heat-killed *S. aureus*[52]. Stimulation of epithelial cells by α-hemolysin could result from its pore-forming activity and the osmotic stress that ensues [37,53].

The response of bMEC to SaS was not the result of an overwhelming cytotoxicity, because the early response was an increase of the reduction activity (AlamarBlue), in keeping with the early overexpression of the cytochrome genes *cyp1a1* and *cyp1b1* induced by killed *S. aureus* or *S. aureus* supernatant [14,17]. Nevertheless, toxicity is likely to have contributed to shaping the response of bMEC to SaS. Apart from genes linked to oxidative stress, genes associated with cyclins and the cell cycle were differentially expressed after exposure to SaS (Table 7), which is in keeping with the results obtained after exposure of sheep MEC to *S. aureus* supernatant [17]. This cell response is likely to be a defense response to the activity of cytotoxic components secreted by *S. aureus* and is likely to differ among strains according to their virulence potential.

A previous study based on the use of ovine MEC cultured under conditions comparable to those of the present study allowed the comparison of the trancriptomic profiles of MEC exposed to either live *S. aureus* or *S. aureus* culture supernatant [17]. This study showed that almost half (286/646) of the DEG were shared between the two stimuli. Nevertheless, the greater difference in response to the two stimuli was manifested at the earlier stimulation time (1 h) and not at 5 h of stimulation. The different physical characteristics of the stimuli (particulate *versus* soluble) may play a role in these observed early differences.

Exposure of bMEC to LPS or SaS induced the differential expression of hundreds of genes (Table 3). Transcriptional profiling studies have demonstrated that cells implicated in immune defenses (including epithelial cells) respond to bacterial stimuli with common transcriptional activation programs, which are interpreted as generic “alarm signals” for infection [54]. Variations on this common transcriptional theme result from cell type-specific and pathogen-specific responses. The initial common host response is largely characterized by features of the innate immune response [55]. The common host transcriptional response includes genes that mediate inflammation, genes that regulate inflammation, genes that activate the local innate defenses, genes that activate the systemic immune response, and genes that limit the immune response. All of these categories were represented in the set of differentially expressed genes by bMEC in response to either LPS or SaS (Tables 4 and 5). An example is the complement factor B, an important component of the alternative pathway of complement activation, which was one of the most up-regulated genes by both LPS and SaS (Tables 4 and 5). Up-regulation of components of the alternative pathway of complement has been reported by others [11], and is in keeping with the capacity of this pathway to operate in milk [56].

In previous publications reporting the response of bMEC to *E. coli* or *S. aureus* or TLR agonists such as LPS or LTA, innate immune genes were among the most numerous and most up-regulated genes [10,11,45]. TLR signaling via Myd88 activates two major pathways leading to transcriptional activation in the nucleus: the NF-κB pathway and the mitogen-activated protein kinase (MAPK) cascade. The intensity and duration of this activation has to be controlled to avoid excessive inflammatory tissue damage, and a range of regulatory factors are set in motion to restrict activation. It is thus not unexpected that *NFKBIA* transcription was increased by LPS and SaS (Tables 4 and 5). It has been shown that activation of the NF-κB protein complex closely correlates with the transcriptional level of *NFKBIA*[57]. *NFKBIA*, which encodes IκBα, is involved in the negative regulation of NF-κB transcription factors. LPS induces the degradation of the negative regulator IκBα, allowing NF-κB to translocate in the nucleus, which in turn triggers a negative feedback loop by promoting the resynthesis of IκBα [58]. In the same vein, it is noteworthy that *DUSP1* was up-regulated in MEC after stimulation with SaS (Table 5). The termination of MAPK activity largely relies on dual specificity phosphatases (DUSP), whose prototypic member, DUSP1, has been shown to be expressed in several cell types upon stimulation with LPS or peptidoglycan, and to contribute to the control of inflammation [59,60]. The up-regulation of *fos* and *jun* by SaS (Table 5) at 3 h and 6 h post-stimulation was remarkable, inasmuch as LPS down-regulated *fos* at 6 h post-stimulation (Table 4). The *fos* gene encodes a leucine zipper protein that can dimerize with proteins of the Jun family, thereby forming the transcription factor complex AP-1. *Staphylococcus aureus* virulence factors were shown to induce *c-fos* expression [61]. The expression of ELRCXC chemokines by human mammary epithelial cells is mainly correlated to the AP-1 pathway and to a lesser extent to the NF-κB pathway [62], which is in line with the stimulated expression of CXCL1, CXCL2, CXCL3, CXCL5 and CXCL8 by SaS (Table 5). An early activation of NF-κB has been evidenced in mouse mammary glands after inoculation with *E. coli*, and molecular imaging showed that this activation occurred first within the mammary epithelium [63]. Induction of NF-κB components and of *NFKBIA* was also reported early (4 h) after infusion of LPS into mouse mammary glands [64]. Thus the results obtained in vitro with bovine MEC are corroborated by data obtained in vivo with mouse mastitis models.

A major finding of the microarray analysis was that several genes of the type I IFN cascade were up-regulated following stimulation with LPS (Table 4), whereas none of these genes were up-regulated following stimulation with SaS (Table 5). In macrophages, TLR2 agonists induce a subset of TLR4-inducible proinflammatory genes, which suggests the use of differential signaling pathways [65]. TLR2 agonists poorly induce IFN-β. LTA in particular did not induce IFN-β expression [66]. By contrast, the LPS-induced TLR4 signaling cascade comprises two pathways: a MyD88-pathway with rapid activation of NF-κB and MAPK, and a MyD88-independent pathway leading to the induction of IFN-inducible genes [67]. A considerable part of the gene expression signature in LPS-stimulated cells depends on Interferon-stimulated genes (ISG) [43]. IFN induction plays an important role in activating the full NF-κB transcriptional response because of transcriptional cooperation between the IRF and NF-κB [58]. A good example of an IFN-dependent gene is *Nos2*, because full transcriptional induction of the *Nos2* gene encoding the inducible nitric oxide synthase (iNOS) requires type I IFN signaling and additional signals emanating from pattern recognition receptors [68,69]. Up-regulation of *Nos2* by staphylococcal LTA occurs, driven by the stimulation of the receptor for the platelet activating factor (PAFR) [66], but LTA did not activate PAFR on bMEC [33]. This is consistent with our observation that *Nos2* was much more differentially expressed by LPS-stimulated than by SaS-stimulated MEC (Figure 5c). An increase in *Nos2* expression in mammary tissue has been reported following infusion with LPS [70].

A consequence of type I IFN cascade triggering by LPS but not by SaS was the differential expression of a number of chemokines encoded by type I IFN responsive genes. These chemokines are dependent on the type I IFN pathway for optimal production. The chemokines CXCL10, CXCL11, CCL2 and CCL5, which are part of the “type I IFN chemokine signature”, mainly attract monocytes, natural killer cells and activated lymphocytes [71,72]. Apart from mononuclear leucocytes, CXCL10, which was highly up-regulated by LPS but not by SaS (Figure 5b), is also implicated in the recruitment and activation of neutrophils at sites of infections in mice and humans [73,74]. Of note, CXCL10 has been found among the highest upregulated genes in the mammary gland of mice 4 h after infusion with *E. coli* LPS [64]. The chemokine CCL20, which attracts memory T lymphocytes and immature DC, depends on NF-κB and type I IFN pathway for full expression [75]. Our RT-qPCR experiments indicated that *Ccl2*, *Ccl5* and *xCcl20* were more up-regulated by LPS than by SaS (Figure 4). A higher expression of *Ccl5* following stimulation of bMEC with *E. coli* than with *S. aureus* has been reported previously [76]. The differential expression of these IFN-regulated chemokines may entail a stronger recruitment of monocytes and lymphocytes in the mammary tissue and milk with LPS than with SaS. More generally, Type I IFN signaling is considered crucial for host resistance against different pathogens, including extracellular bacteria such as *E. coli* and *Streptococcus agalactiae*[77], although the end-result can be favorable or detrimental to the host, depending on the circumstances [42].

Several studies have demonstrated that *S. aureus* activates IFN-dependent genes [25,78]. The capacity to activate the type I IFN pathway may be linked to the expression of virulence genes by live *S. aureus*[25], because killed *S. aureus* do not induce an inflammatory response as strong as do live bacteria [79,80]. Some expression of the type I IFN pathway upon stimulation of MEC with SaS could have been expected, because SpA was found in SaS, and SpA is able to activate the type I IFN pathway in airway epithelial cells [81]. The SaS used for our experiments was shown to contain SpA and alpha hemolysin, yet there was no indication that the type I IFN pathway was activated. There may even be inhibition of IFN-β activity on bMEC by SaS (Figure 5). Activation of IFN-dependent genes may also result from the autocrine/paracrine stimulation of MEC by the IL-6 and downstream triggering of the STAT3 signaling pathway as hypothesized by others [14]). The lack of overexpression of IFN-dependent genes by *S. aureus* culture supernatant or live *S. aureus* by ovine MEC or human respiratory cells has also been reported [16,17]. In the study by Günther et al. [14] involving bMEC, it is of note that overexpression of IFN-dependent genes was detected after 24 h of exposure to heat-killed *S. aureus*. Another group reported the overexpression of *Ccl5* and *Cxcl10* only after 24 h of exposure of bMEC to heat-killed *S. aureus*[44]. Thus, IFN-dependent gene stimulation could be a late event in *S. aureus*/MEC interaction, on the contrary to the *E. coli*/MEC interaction.

Bovine MEC readily produce chemokines in response to bacterial stimuli, and apparently there is a good correlation between mRNA levels and protein secretion [22,33]. On the contrary, increases in TNF-α mRNA transcripts do not imply protein production by bMEC, as shown previously [33,51] and in this study. In addition to being regulated at the transcriptional level, TNF-α production is subject to translational control. In particular, the 3′-untranslated region (UTR) of TNF-α mRNA contains an AU rich sequence that imposes a translational block on the mRNA, and this block is lifted only in cells able to produce TNF-α in response to the appropriate stimuli [82,83]. Production of TNF-α by mouse or bovine MEC has been reported after stimulation with LPS or live *S. aureus*, but the secretion was modest, less than 50 pg/mL [15,22,84]. It may be essential for the mammary gland to keep the expression of TNF-α under tight control, because one autocrine effect of this cytokine is to inhibit the synthesis of caseins by MEC [85]. Secretion of IL-1β by bMEC has not been reported to date, although increases in transcript numbers following stimulation with LPS or LTA is documented [10,11,76]. Maturation and secretion of IL-1β is known to be dependent on the activation of the inflammasome and of caspase-1 [86], events that have not been documented in bMEC.

A functional pathway most affected by SaS was the IL-17A transduction pathway (Table 7). This does not mean that IL-17A directly contributed to the response of MEC to SaS, but that MEC overexpressed several genes associated with this pathway. We have recently shown that bovine MEC respond to bovine IL-17A by producing chemokines and overexpressing a number of genes related to innate immune defenses [51], and up-regulation of the gene coding IL-17A in milk leukocytes from cows suffering from *S. aureus* mastitis has been reported [87,88]. These observations prompt further studies to investigate the role of IL-17A in the response of the mammary gland to infection.

**Figure 6:**
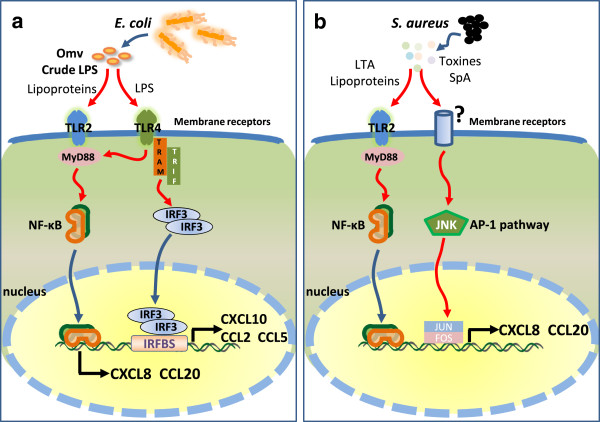
**Main signaling pathways supposed to be activated in MEC exposed to either *E. coli *crude LPS (a) or *S. aureus *culture supernatant (b).** Crude LPS (a simplified substitute of outer membrane vesicles, omv) is sensed by both TLR2 and TLR4, which activates the NF-κB pathway by the MyD88-dependent pathway. In addition, the TRAM-TRIF-IRF3 pathway leads to the activation of genes that have an IRF-binding site in their promoter sequence. *S. aureus* culture supernatant (SaS) is sensed by TLR2 and other unidentified receptors that activate the NF-κB and AP-1 pathways. As a result, *E. coli* stimulation induces a higher number of genes (IFN-stimulated genes) than does *S. aureus* stimulation. This is exemplified by the overexpression of a panel of chemokine genes that have the potential to recruit a greater variety of leukocytes (see text). LTA: lipoteichoic acid; MyD88: myeloid differentiation primary-response gene 88; TRIF: TIR-domain-containing adaptor protein inducing IFN-β; TRAM: TRIF related adaptor molecule; IFR3: IFN regulatory factor 3; IRFBS: IRF-binding site; JNK: Janus kinase; AP-1: activator protein 1, composed of the Jun and Fos proteins.

Inflammatory responses to *E. coli* and *S. aureus* mastitis differ in several ways. A major difference is the intensity of the systemic and local responses, which is considered to result from underlying differences in the magnitude of production of key pro-inflammatory cytokines and chemokines [89]. In particular IL-1β is found in greater concentration in milk of *E. coli* mastitis than in milk of *S. aureus* mastitis, and TNF-α is found in bovine milk in case of *E. coli* but not *S. aureus* mastitis [5,6]. The inability of bMEC to secrete TNF-α and IL-1β in response to crude LPS or SaS suggests that these cells may not be responsible for the difference in production of these pro-inflammatory cytokines. These cytokines are likely to be produced by resident or recruited leucocytes rather than by MEC. The case of CXCL8 is different, because the higher concentrations found in milk of *E. coli* than *S. aureus* mastitis were mirrored by a higher production by bMEC in this study. Given that milk concentrations detected during the course of *E. coli* intramammary infection are comparable with those detected in response to intramammary infusion of LPS [89], this suggests that MEC contribute to this difference. Several studies suggest that MEC contribute to the production of chemokines found in milk of mammary tissue in the course of mastitis, even though most of the results are based on mRNA rather than on protein quantifications [8,10,12,13,22,33,90]. In this study we found that the chemokine profile induced by the stimulation of bMEC by LPS and SaS differ in nature and magnitude. This differential expression may result in part from the differential expression of IFN-inducible genes, which clearly sets apart the early responses of bMEC to LPS and SaS (Figure 6). A consequence of this stimulation of IFN-inducible genes is the differential expression of a number of downstream genes such as certain chemokines (*Ccl2*, *Ccl5*), pro-inflammatory genes (*IL6*) and innate immune defense genes (*Nos2a*). This in turn is likely to have a strong influence on the inflammatory and immune responses to the corresponding pathogens, *S. aureus* and *E. coli*. In particular the wider set of chemokines induced by LPS is likely to recruit a wider diversity of leucocytes than could do *S. aureus* MAMP. Also, self-defense of the epithelium may be more stimulated by *E. coli* than by *S. aureus* MAMP. These features could contribute to the different clinical manifestations and outcome of mastitis caused by these two pathogens. By combining in vitro and in vivo approaches and using a variety of models, an improved understanding of the complex interactions of pathogens with the mammary gland can be anticipated.

## Competing interests

The authors declare that they have no competing interests.

## Authors’ contributions

PR and FBG conceived the study. RR, KJ and EG participated in its design and coordination. GF contributed to the IFN studies. PC and FBG performed most the experiments. KJ and EG carried out the microarray assay. RR and CRG analyzed the microarray data. PR drafted the final version of the manuscript. All authors corrected and approved the final manuscript.

## Supplementary Material

Additional file 1 **Selection of culture duration and *S. aureus *strain for production of SaS. a**) Monitoring of protein secretion by *S. aureus* N305 as a function of culture duration (hours) in DMEM/F12 cell culture medium. Bacteria were grown at 37°C in RPMI 1640/DMEM (1:1) medium for the indicated times, before analysis by SDS-PAGE. **b**) Concentration of CXCL8 in culture supernatant of bMEC exposed to SaS for 8 h was determined by ELISA. Results are means of a duplicate culture of cells from one cow. Click here for file

Additional file 2 **Schematic representation of the microarray experimental design.** Gene expression in bMEpC samples collected at 3 or 6 h after exposure to either SaS or LPS was analyzed by comparison with samples collected at 3 or 6 h without exposure to stimuli. Each arrow represents one microarray slide with the direction indicating the cDNA labelling from Cy5 to Cy3-labelled cDNA. Click here for file

Additional file 3 **Evaluation of the toxic effect of SaS on bMEC.** Chemical reduction of growth medium supplemented with AlamarBlue by bMEC after 3 h and 6 h of exposure to 25% N305 SaS. Results are means from bMEC of 5 cows. Click here for file

Additional file 4 **List of differentially expressed genes.** Bovine MEC were stimulated with crude LPS or SaS for 3 h or 6 h, and the reaction of the cells was investigated through gene expression profiling by microarray analysis. Differential expression relative to unstimulated cells (Fold changes) are shown after a 3 h-exposure to LPS (LPS3h_vsREF), 6 h-exposure to LPS (LPS6h_vsREF), 3 h-exposure to SaS (SA3h_vsREF), and 6 h-exposure to SaS (SA6h_vsREF). Click here for file
