# Supplementary figures and images for: Differential response of bovine mammary epithelial cells to Staphylococcus aureus or Escherichia coli agonists of the innate immune system
Source: Vet Res. 2013 Jun 11;44(1):40. doi: 10.1186/1297-9716-44-40 (PMC3686618; doi:10.1186/1297-9716-44-40)

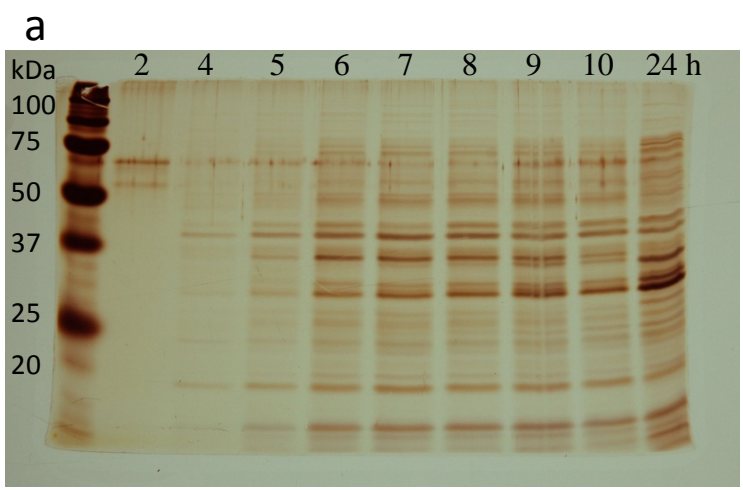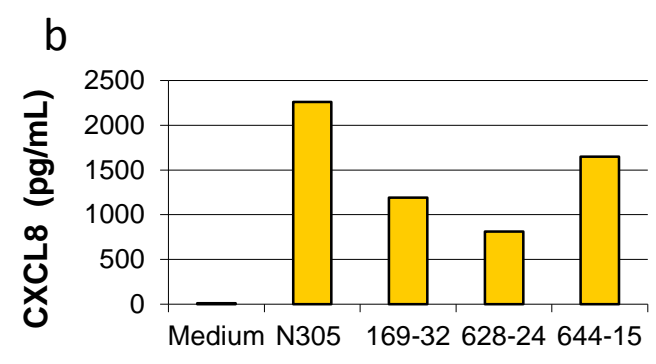

Supplement: Additional file 1 — Selection of culture duration and S. aureus strain for production of SaS. a) Monitoring of protein secretion by S. aureus N305 as a function of culture duration (hours) in DMEM/F12 cell culture medium. Bacteria were grown at 37°C in RPMI 1640/DMEM (1:1) medium for the indicated times, before analysis by SDS-PAGE. b) Concentration of CXCL8 in culture supernatant of bMEC exposed to SaS for 8 h was determined by ELISA. Results are means of a duplicate culture of cells from one cow. [file 1297-9716-44-40-S1.pdf]

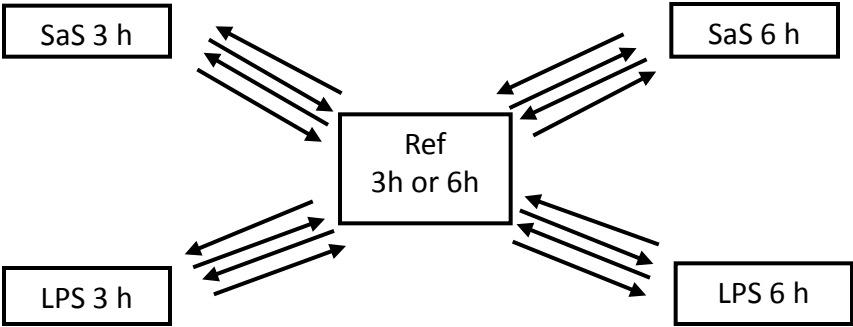

Supplement: Additional file 2 — Schematic representation of the microarray experimental design. Gene expression in bMEpC samples collected at 3 or 6 h after exposure to either SaS or LPS was analyzed by comparison with samples collected at 3 or 6 h without exposure to stimuli. Each arrow represents one microarray slide with the direction indicating the cDNA labelling from Cy5 to Cy3-labelled cDNA. [file 1297-9716-44-40-S2.pdf]

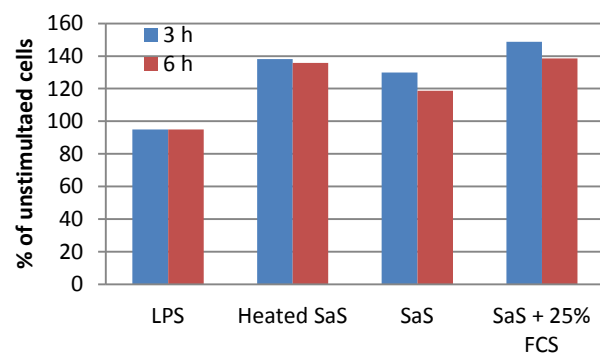

Supplement: Additional file 3 — Evaluation of the toxic effect of SaS on bMEC. Chemical reduction of growth medium supplemented with AlamarBlue by bMEC after 3 h and 6 h of exposure to 25% N305 SaS. Results are means from bMEC of 5 cows. [file 1297-9716-44-40-S3.pdf]
